# Difference in Performance of EPI Pigs Fed Either Lipase-Predigested or Creon®-Supplemented Semielemental Diet

**DOI:** 10.1155/2021/6647734

**Published:** 2021-07-08

**Authors:** Stefan G. Pierzynowski, Anna Socha-Banasiak, Monika Sobol, Grzegorz Skiba, Stanisława Raj, Olena Dovban, Galyna Ushakova, Jarosław Woliński, Nadiia Mosiichuk, Paulina Szczurek-Janicka, Marek Pieszka, Marian Kamyczek, Ewa Święch, Halyna Shmigel, Marcin Sonta, Elżbieta Czkwianianc, Kateryna Pierzynowska

**Affiliations:** ^1^Department of Biology, Lund University, Lund 22362, Sweden; ^2^SGP+Group, Trelleborg 23132, Sweden; ^3^Department of Biology, Institute Rural Medicine, Lublin 20950, Poland; ^4^Department of Gastroenterology, Allergology and Pediatrics, Polish Mother's Memorial Hospital Research Institute, Łódź 93-338, Poland; ^5^Laboratory of Large Animal Models, Kielanowski Institute of Animal Physiology and Nutrition, Polish Academy of Sciences, Jabłonna 05-110, Poland; ^6^Department of Animal Nutrition, Kielanowski Institute of Animal Physiology and Nutrition, Polish Academy of Sciences, Jabłonna 05-110, Poland; ^7^Department of Biochemistry and Physiology, Oles Honchar Dnipro National University, Dnipro 49050, Ukraine; ^8^Department of Animal Physiology, Kielanowski Institute of Animal Physiology and Nutrition, Polish Academy of Sciences, Jabłonna 05-110, Poland; ^9^Department of Biochemistry and Biotechnology, Vasyl Stefanyk Precarpathian National University, Ivano-Frankivsk 78018, Ukraine; ^10^Department of Nutrition Physiology, National Research Institute of Animal Production, Balice 32-083, Poland; ^11^National Research Institute of Animal Production, Experimental Station Pawłowice, Pawłowice 64-122, Poland; ^12^Department of Animal Breeding, Institute of Animal Sciences, Warsaw University of Life Sciences—SGGW, Ciszewskiego 8, 02-786 Warsaw, Poland

## Abstract

Pancreatic enzyme replacement therapy (PERT) and fat predigestion are key in ensuring the optimal growth of patients with cystic fibrosis. Our study attempted to highlight differences between fat predigestion and conventional PERT on body composition of young pigs with exocrine pancreatic insufficiency (EPI). EPI and healthy pigs were fed with high-fat diet for six weeks. During the last two weeks of the study, all pigs received additional nocturnal alimentation with Peptamen AF (PAF) and were divided into three groups: H—healthy pigs receiving PAF; P—EPI pigs receiving PAF+PERT; and L—EPI pigs receiving PAF predigested with an immobilized microbial lipase. Additional nocturnal alimentation increased the body weight gain of EPI pigs with better efficacy in P pigs. Humerus length and area in pigs in groups L and P were lower than that observed in pigs in group H (*p* value 0.005-0.088). However, bone mineral density and strength were significantly higher in P and L as compared to that of H pigs (*p* value 0.0026-0.0739). The gut structure was improved in P pigs. The levels of neurospecific proteins measured in the brain were mainly affected in P and less in L pigs as compared to H pigs. The beneficial effects of the nocturnal feeding with the semielemental diet in the prevention of EPI pigs' growth/development retardation are differently modified by PERT or fat predigestion in terms of growth, bone properties, neurospecific protein distribution, and gut structure.

## 1. Introduction

Diseases that are associated with loss or block of the pancreatic parenchyma function (e.g., cystic fibrosis (CF), obstruction of the main pancreatic duct, extensive necrotizing acute pancreatitis, pancreatic cancer, and Shwachman-Diamond syndrome) may lead to exocrine pancreatic insufficiency (EPI), which results in the malabsorption of nutrients, vitamin deficiencies, poor growth, delayed puberty, weight loss, and an increased risk of death [1].

CF patients usually exhibit general and skeletal growth retardation [2]. CF-related bone disease is common among adolescents and adult patients [3], which is the primary reason for the recommendation of early (≥8 years old) and periodic (every 1-5 years) bone health assessments in CF patients, using dual-energy X-ray absorptiometry (DXA) [4].

Oral pancreatic enzyme replacement therapy (PERT) is recommended to EPI patients [4]. Some patients, however, do not respond well to enzyme supplementation. The poor response of some patients to PERT may be due to the autoinactivation of the enteric-coated enzymes, directly after reaching the gut [5]. For this reason, alternate strategies of replacement therapy are being examined, including predigestion using immobilized lipase to improve fat digestion and absorption, in order to obtain satisfactory clinical responses [6].

In patients with CF obtaining PERT, when the consumption of a high energy diet does not ensure adequate nutritional status, enteral nasogastric tubes or gastrostomy feeding strategies are advised. In these cases, nocturnal feeds with a high energy polymeric formula are recommended [4]. Semielemental feeds, containing peptides and medium-chain fatty acids (MCFAs), without enzyme replacement [6] lead to the same level of improvement in fat absorption and growth as that of a polymeric formula with PERT [7]. It is worth noticing that none of the currently available PERT formulations are indicated for nocturnal feedings, so many EPI patients receiving semielemental nocturnal feedings supplemented with currently available PERT formulations continue to struggle nutritionally and experience clinical symptoms related to malabsorption of fats, being not able to increase actual body weight gain [8].

Considering the facts mentioned above and that immobilized lipase has been shown to play an essential role in the efficient digestion of fat in order to ensure optimal growth and development [8], we wanted to prove this experimentally. The aim of the present study was to highlight the beneficial effects of semielemental diet feeding, as well as PERT vs. fat predigestion on the growth, body composition, distribution of neurospecific proteins, and gut structure of young, growing EPI pigs.

## 2. Materials and Methods

The present study was carried out in strict accordance with the recommendations in the *Guide for the Care and Use of Laboratory Animals* of the National Institutes of Health. The experimental procedures used in the current study were approved by the II Local Ethics Committee on Animal Experimentation of the Warsaw University of Life Sciences, Poland (decision number: WAW2/15/2017). All efforts were made to minimize animal suffering during experimental procedures.

### 2.1. Animals, Housing, and Diets

The study was carried out on thirty-two weaned piglets (Line 990, National Research Institute of Animal Production, Experimental Station Pawłowice, Poland). From postnatal day 28 to 35, pigs were accustomed to the housing and feeding conditions and were fed a high-fat diet (20% fat content, HF20, Table 1). On postnatal day 35, eight randomly selected pigs were allocated to the healthy group (group H), while the remaining pigs underwent pancreatic duct ligation surgery [9] in order to induce EPI. Following surgery, all pigs (healthy and operated) were fed a high-fat diet (25% fat content, HF25) which was composed of HF20 diet and cream (36% fat content) in a proportion of 11 : 5. Pigs were offered food twice daily (between 8 AM and 9 AM and between 2 PM and 3 PM), in an amount of 2.0% of their body mass per meal (160 kcal/kg bwt/day), for a period of four weeks.

Following four weeks of high-fat diet feeding (on postnatal day 63), all pigs that underwent pancreatic duct ligation surgery were assessed for the presence of standard EPI signs, which include growth retardation, maldigestion, and voluminous stools. Following an overnight fast, the pigs with EPI were randomly divided into two groups (P (11 pigs) and L (13 pigs)). From postnatal days 64 to 74, all pigs consumed a HF25 diet twice daily.

Each evening (between 8 PM and 12 PM), the pigs received additional nocturnal enteral feeding (total amount of 400 mL (48 kcal/kg bw/day), in a dose of 50 mL every 30 minutes) via a gastric tube (G-tube): Peptamen AF (Nestlé Health Science, Stockholm, Sweden) (without additional enzymes (group H)) or with enteric-coated pig pancreatic enzymes (Creon 25000, 4 capsules at the start of the intragastric additional nocturnal feeding and 4 capsules at the end of the nocturnal feeding)—group P; Peptamen AF with an immobilized microbial lipase (lipase 534641, Sigma-Aldrich) as a functional nutritional drinking formula—group L. In order to feed pigs from group L, immobilized microbial lipase (iML, 5 g/2 l Peptamen AF) in a mesh bag was placed into the Peptamen AF mixture and mixed with a stirrer for 15 min, at a temperature of between 35 and 37°C, before each feeding [10]. The absence of lipase activity in the predigested formula was confirmed using a lipase activity assay (lipase activity assay kit, MAK 046, Sigma-Aldrich Sweden AB, Stockholm, Sweden). The level of Peptamen predigestion was controlled by nonesterified fatty acid (NEFA) content measurement. The NEFA level was determined using a standard colorimetric kit (NEFA-C kit, Wako Chemicals GmbH, Neuss, Germany).

Throughout the study, the pigs were housed individually in pens (3.3 m^2^) equipped with nipple drinkers, on a concrete floor without straw. Pigs had olfactory, auditory, and visual contact with each other. The environmental conditions in the piggery (air temperature: 18–20°C, relative humidity: 60–70%, and air flow: 0.2–0.4 m/s) were regulated by a Fancom ventilation system (Fancom BV, NK Panningen, The Netherlands, model ISM.12). A 12-hour day-night cycle, with lights on from 06:00 AM to 6:00 PM, was applied.

### 2.2. Densitometry Analysis of the Pigs' Body

At the end of the study (postnatal day 75), each pig was scanned using DXA (Norland XR-800™ densitometer scanner with a “Whole-Body” scan type, Norland, A Cooper Surgical Company, Fort Atkinson, WI, USA). The following measurements were determined: lean mass (kg), fat mass (kg), fat percentage, bone area (cm^2^), bone mineral content (BMC, g), and bone mineral density (BMD, g/cm^2^). Before scanning, pigs were subjected to short-lasting sedation by injection using a mixture of ketamine hydrochloride (2 mg/kg body weight) and xylazine (0.2 mg/kg body weight). The pigs were then placed in a ventral position with all limbs extended. The DXA scans were obtained using standard procedures, as described by the manufacturer, for scanning and analysis. Two scans were performed on each pig.

### 2.3. Euthanasia and Sample Collection

After DXA scanning, the pigs were sedated using azaperone (Stresnil, LEO, Helsingborg, Sweden), 5 mg/kg body weight, and euthanized using a single dose of i.v. injected sodium pentobarbiturate (100 mg/kg body weight). The brain and gastrointestinal tract were then dissected out. The cerebellum and hippocampus were isolated immediately at 4°C and frozen until further analysis. Segments of the middle jejunum (15 mm long) were dissected, and samples of each section were collected and immediately fixed in a 10% neutral formalin solution. After the 24-hour fixation period, the intestinal samples were routinely embedded in paraffin. From each left half-carcass, the humerus was manually dissected. Following excision, the bones were cleaned of any remaining flesh and stored at −20°C until further BMD analysis.

### 2.4. Gut Structure Analysis

The paraffin-embedded samples were cut into 4.5 *μ*m sections and applied to saline-treated slides. Next, the sections were dewaxed in xylene and rehydrated in decreasing grades of ethanol and then stained with haematoxylin and eosin. Three slides were randomly selected for each intestinal section, and 30 measurements of the muscularis layer were then performed, using a light microscope (Axioskop 40, Zeiss, Germany), coupled with computer software for image analysis (Axio Vision 4.2 Release, Zeiss, Germany).

### 2.5. Humerus Morphometry, Densitometry, and Mechanical Properties

The humeri were thawed at room temperature (23°C) for 12 h prior to use. The weight and length of the bones were measured, and the bones were then scanned using DXA (Norland XR-800™ densitometer scanner with a “research” scan type, Norland, A Cooper Surgical Company, Fort Atkinson, WI, USA). During scanning, each bone was positioned horizontally, with the bone head facing upwards and the condyles downwards and scanned from the distal to the proximal end. All scans were performed in triplicate in order to avoid any rotation of the bone, since inconsistencies in their orientation could adversely affect test precision. All scans were performed by the same operator. Values of BMC (g) and BMD (g/cm^2^) were recorded. After scanning, the three-point bending test was performed to determine the mechanical bone characteristics using a TA-HDi Texture Analyser (Godalming, Surrey, UK) with a head speed of 1 kN, 10 mm/min. The value of maximum strength (kg) was determined. The distance between supports of the bone was set at 40% of the bone length and the measuring head loaded bone samples at the midshaft with a constant speed of 50 mm/min.

### 2.6. The Analysis of Neurospecific Protein Distribution

Tissues were homogenized and processed as previously described [11]. The concentrations of glial fibrillary acidic protein (GFAP) and neural cell adhesion molecule (NCAM) in the fractions were determined using ELISA as previously described [12]. Optical density was measured using an Anthos-2010 absorbance reader (Anthos Labtec Instruments GmbH, Wals-Siezenheim, Austria).

### 2.7. Statistical Analysis

Statistical analyses were performed using Statistica software (version 12, StatSoft Tulsa, OK, USA). With an *α* level of 0.05, power established at 80%, and an effect size of 0.85, the required total sample size was 24 (i.e., *n* = 8/group). The hypothesized effect size of 0.85 was calculated from the descriptive statistics of a previous study [13]. Data are presented as means and standard deviations (SD). Obtained data were analysed using a one-way ANOVA followed by a Tukey post hoc test for multiple comparisons. Statistical significance was set at *p* < 0.05.

## 3. Results

### 3.1. Body Weight Gain and Feed Intake

During the study, feed intake was not different between groups. There were no significant differences in body weight of the pigs between groups at the beginning of the study (on postnatal day 28) and after the accommodation period (on postnatal day 35). Four weeks after pancreatic duct ligation, the body weight of the pigs in groups P and L was significantly lower compared to that of the pigs in group H (*p* < 0.05, Table 2). The body weight gain of pigs in groups P and L prior to commencement of the treatment (between postnatal days 35 and 64) was equal and amounted to 30% and 26% of that observed in the pigs in group H, respectively. After introduction of the additional nocturnal alimentation, the total body weight gain increased to up to 64% and 49% in pigs in groups P and L, respectively, and of that of group H pigs, with the increase in body weight gain being lower in L compared to P pigs.

### 3.2. Whole-Body Composition and Bone Properties

Pigs in groups P and L had significantly lower whole-body lean mass (*p* < 0.01), fat mass (*p* < 0.001), and body fat mass percentage (*p* < 0.001, Table 3) compared with those in group H. The whole-body bone area differed only between pigs in groups L and H (*p* < 0.05); however, the whole-body BMC and BMD were not different among groups.

The humeri of pigs in groups P and L had a similar mass, length, area, BMC, and BMD (and strength (Table 3). The mass of the humeri differed only between pigs in groups L and H (*p* < 0.05). Humerus length and area in pigs in groups L and P were lower than that observed in pigs in group H (*p* value ranged from 0.005 to 0.088). However, BMD and strength were significantly higher in P and L as compared to that of H pigs (*p* value ranged from 0.0026 to 0.0739).

### 3.3. Gut Morphology

**Figure 1:**
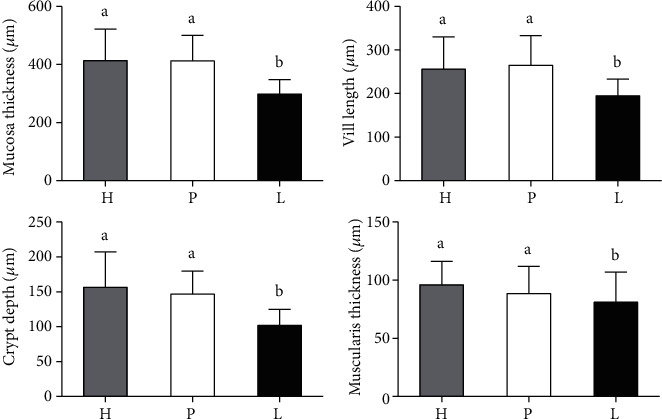
Histomorphometry (mucosa thickness, villi length, crypt depth, and muscularis thickness) of the middle part of the jejunum at the end of the study on healthy pigs fed a HFD and EPI pigs fed a HFD either with PERT or with microbial lipase, with all pigs receiving additional nocturnal feeding with Peptamen AF (PAF). H: healthy pigs+PAF; P: EPI pigs+PAF enriched with enteric-coated pig pancreatic enzymes; L: EPI pigs+PAF predigested with microbial lipase. Data are presented as the mean ± SD. Small letters given with result bars describe significant differences when *p* < 0.05.

Histological analysis (Figure 1) of the middle part of the jejunum showed a decrease (*p* < 0.0001) in all the parameters investigated in pigs from group L compared to those observed in groups H and P, which were not different from one another.

### 3.4. The Distribution of Neuron and Astrocyte-Specific Proteins

The analysis of neurospecific protein distribution revealed a significant decrease in the soluble NCAM level in the cerebellum of P pigs compared to such observed in the H pigs. At the same time, a significant increase in the level of soluble NCAM was observed in the hippocampus of both group P and group L pigs compared to the values obtained for group H pigs (Table 4).

A significant decrease in the level of soluble GFAP in both the hippocampus and cerebellum was noted in group P pigs in comparison to group H pigs. At the same time, levels of soluble GFAP were significantly lower in the hippocampus, but not in the cerebellum of group L animals when compared to healthy pigs (group H). The filamentous GFAP level was significantly increased in the cerebellum and decreased in the hippocampus in the group P pigs compared to the group H animals (Table 4).

## 4. Discussion

### 4.1. Body Weight Gain and Gut Structure Analysis

It has previously been shown in the porcine EPI model, that a lack of pancreatic enzyme secretion reduces digestion and contributes to overall growth retardation [9], while pancreatic enzyme supplementation leads to an increase in the body weight of EPI pigs [14]. In general, the results of the present study confirm the observations listed above. However, our data also shows that body weight gain is different in pigs receiving the additional nocturnal alimentation with a semielemental diet together with PERT (group P), versus those receiving the semielemental diet predigested with microbial lipase (group L), for a period of 10 days. The body weight gain of pigs in group P was significantly higher than that observed in group L pigs. Thus, the predigestion of the fat in the semielemental diet by lipase (group L) was less efficient than digestion by the combined action of the enteric-coated pig pancreatic enzymes (group P). The most probable explanation for this phenomenon is the fact that Peptamen AF is only a semielemental formula and the pancreatic enzymes other than lipase (proteinase+amylase) in the PERT can further digest the formula polymer ingredients, thus making them more and more available for absorption.

Previous studies also observed adverse changes in intestinal parameters in EPI pigs [13]. Moreover, abnormalities in the intestinal structure correlated with the age of EPI development [15]. Previous research by our lab [10] allowed us to hypothesize that immobilized lipase supplementation may influence histomorphometric parameters and in turn improve nutrient availability. The data obtained from the present study showed that only treatment with the enteric-coated pig pancreatic enzymes (group P), and not with microbial lipase (group L), was able to improve the intestinal parameters. These results could also possibly be explained by the role of the proteases in the regulation of the maturation and modelling of the gut epithelium [16].

### 4.2. Whole-Body Composition and Bone Properties

In the paediatric population, lean body mass and BMC have been shown to be more sensitive indicators of nutritional deficits than a low body mass index (BMI) [4]. In the present study, we observed a reduction in lean body mass, fat mass, and whole-body fat percentage in pigs with EPI compared to healthy pigs. This data corroborates results obtained by other authors in their investigations of the effects of EPI on body composition [17]. Since we found no differences between alternative (group L) and standard methods of therapy (group P), this may indicate an overall effect of fat absorption, regardless of the treatment type, on whole-body composition.

BMD has been proven to be dependent on the bone region being studied [18, 19]. In the current study, humerus length and area were lower in pigs from groups obtaining enzyme supplementation compared to that observed in group H. It has been reported that EPI results in decreased IGF-1 serum levels that are in turn associated with impaired growth [13]. Surprisingly, in the current study, we found that the BMD and maximum strength of humeri in pigs with EPI were significantly higher than those of the healthy pigs. This interesting observation may possibly be explained by the decreased bone mass and unchanged mineral content observed in the femur of EPI pigs.

### 4.3. Neurospecific Protein Distribution

In the present study, we observed an increase in the level of soluble NCAM of up to 53% in the hippocampus of group P pigs, as well as in group L pigs, compared to that of group H pigs. Our current data concurs with previous observations from our lab [10], and the observed increase in the soluble NCAM level could be recognized as a consequence of EPI development, which is known to cause neurological deficits [20, 21].

We observed a 16% increase in filamentous GFAP in the cerebellum and a 14% decrease in the hippocampus of group P pigs compared to group H pigs. A decline in filamentous GFAP in the hippocampus of the porcine EPI model has previously been observed, in association with a reduced astrocyte number [20]. Such a decline indicates the depolymerisation of intermediate filaments of the astrocyte cytoskeleton in the EPI pigs receiving no PERT and is observed under chronic stress [22] and associated with depressive disorders [23]. At the same time, an increased level of filamentous GFAP in the cerebellum could be a sign of astrocytic activation which could alter neurotransmission and impact cognitive and motor function [24, 25].

It is worth mentioning that group L pigs showed levels of neurospecific proteins, both in the hippocampus and in the cerebellum, close to those observed in the group H pigs. Thus, predigestion with microbial lipase could help in preventing the development of EPI-related neurological deficits.

## 5. Conclusions

We conclude that the benefits of feeding EPI pigs with semielemental diet can be improved, however, differently, by PERT supplementation or diet predigestion with lipase. The predigestion of the diet with microbial lipase leads to the crucial change in body composition and bone mineralisation in the EPI pig model. However, PERT, which ensures the full spectrum of digestion, has a significantly better effect on body weight gain than that of fat predigestion with lipase.

## Figures and Tables

**Table 1:** Ingredients, chemical composition, and nutritive value of the high-fat diet with 20% fat content (HF20).

Indices	HF20
Ingredients (g/kg)	
Barley	261.00
Wheat	274.80
Soybean meal	200.00
Fish meal (65% crude protein)	20.00
Rapeseed oil	200.00
Premix 0.5% grower^1^	5.00
Monocalcium phosphate	15.00
Fodder chalk	10.00
Fodder salt	2.00
Zinc oxide	0.17
Lysine	5.00
Methionine	2.00
Threonine	2.00
Tryptophan	2.00
Acidifier	1.00

Chemical composition (g/kg)	
Dry matter	905
Ash	24.3
Organic matter	881
Crude protein	166
Ether extract	216
Crude fibre	33.5
Starch	311

Nutritive value (determined) (g/kg)	
Lysine	13.20
Methionine	4.65
Threonine	7.80
Tryptophan	3.90
P	6.97
Ca	9.56
Na	1.14

Metabolisable energy (MJ/kg)	16.8 (4012.7 kcal)
Lysine/metabolisable energy (g/MJ)	0.79

**Table 2:** Body weight (BW) and total weight gain (TWG) of experimental animals.

Item	Group			*p* value		
	H	P	L	P vs. H	L vs. H	L vs. P
BW						
64 days of age	9.85 ± 0.34	8.57 ± 0.30	8.71 ± 0.26	0.0222	0.0465	0.9435
74 days of age	13.73 ± 0.42	11.07 ± 0.38	10.63 ± 0.33	0.0002	0.0010	0.6865

TWG						
35-64 days of age	2.98 ± 0.19	0.90 ± 0.17	0.79 ± 0.15	0.0001	0.0001	0.8964
64-74 days of age	3.88 ± 0.16	2.50 ± 0.14	1.92 ± 0.12	0.0001	0.0001	0.0124
35-74 days of age	6.86 ± 0.31	3.40 ± 0.28	2.71 ± 0.25	0.0001	0.0001	0.1992

H: healthy pigs+PAF; P: EPI pigs+PAF enriched with enteric-coated pig pancreatic enzymes; L: EPI pigs+PAF predigested with microbial lipase. Data are presented as the mean ± SD.

**Table 3:** Whole-body composition and bone properties of experimental animals at the end of the study.

Item	Group			*p* value		
	H	P	L	P vs. H	L vs. H	L vs. P
Whole-body composition						
Lean mass (kg)	11.94 ± 0.36	10.09 ± 0.32	9.71 ± 0.28	0.0014	0.0020	0.6890
Fat mass (kg)	1.84 ± 0.10	0.93 ± 0.09	0.85 ± 0.08	0.0001	0.0001	0.8476
Total fat (%)	12.63 ± 0.65	8.10 ± 0.58	7.23 ± 0.51	0.0001	0.0001	0.5462
Bone area (cm^2^)	705.4 ± 13.02	678.1 ± 11.63	658.3 ± 10.2	0.3060	0.0327	0.4540
BMC (g)	260.8 ± 7.81	256.6 ± 6.98	249.9 ± 6.12	0.9228	0.5898	0.7809

Morphometry, densitometry, and biomechanical properties of humeri						
Mass (g)	58.34 ± 1.97	53.15 ± 1.76	50.52 ± 1.54	0.1554	0.0168	0.5445
Length (mm)	100.99 ± 1.07	96.59 ± 0.95	96.30 ± 0.84	0.0123	0.0070	0.9739
Area (cm^2^)	25.84 ± 0.56	24.13 ± 0.51	23.24 ± 0.44	0.0877	0.0047	0.4314
BMC (g)	10.14 ± 0.40	10.30 ± 0.36	9.73 ± 0.32	0.9590	0.7543	0.5107
BMD (g/cm^2^)	0.391 ± 0.01	0.422 ± 0.01	0.416 ± 0.01	0.0242	0.0739	0.8673
Maximum strength (kg)	80.83 ± 3.51	97.93 ± 3.14	96.09 ± 2.75	0.0026	0.0079	0.9093

H: healthy pigs+PAF; P: EPI pigs+PAF enriched with enteric-coated pig pancreatic enzymes; L: EPI pigs+PAF predigested with microbial lipase. Data are presented as the mean ± SD.

**Table 4:** Neurospecific protein distribution (*μ*g/100 mg of tissue) in cerebellum and hippocampus.

Protein, brain area	Group			*p* value		
	H	P	L	P vs. H	L vs. H	L vs. P
sNCAM, cerebellum	0.52 ± 0.13	0.31 ± 0.11	0.46 ± 0.17	0.049	0.684	0.188
sNCAM, hippocampus	0.40 ± 0.07	0.59 ± 0.15	0.61 ± 0.08	0.014	0.008	0.962
sGFAP, cerebellum	20.48 ± 1.84	18.57 ± 0.68	19.19 ± 0.96	0.030	0.169	0.615
sGFAP, hippocampus	16.46 ± 0.98	13.33 ± 0.84	13.83 ± 2.21	0.004	0.015	0.809
fGFAP, cerebellum	134.70 ± 11.01	155.9 ± 10.75	136.00 ± 3.46	0.012	0.969	0.018
fGFAP, hippocampus	142.90 ± 14.57	84.57 ± 11.18	156.10 ± 14.27	<0.001	0.208	<0.001

sNCAM: soluble neural cell adhesion molecule; sGFAP: soluble glial fibrillary acidic protein; fGFAP: filamentous glial fibrillary acidic protein; H: healthy pigs+PAF; P: EPI pigs+PAF enriched with enteric-coated pig pancreatic enzymes; L: EPI pigs+PAF predigested with microbial lipase. Data are presented as the mean ± SD.

## Data Availability

All data relevant to the study are included in the article.

## Abbreviations

CF: Cystic fibrosis
EPI: Exocrine pancreatic insufficiency
DXA: Dual-energy X-ray absorptiometry
PERT: Pancreatic enzyme replacement therapy
MCFAs: Medium-chain fatty acids
HF20: High-fat diet with 20% fat content
HF25: High-fat diet with 25% fat content
PAF: Peptamen AF
iML: Immobilized microbial lipase
NEFA: Nonesterified fatty acids
BMC: Bone mineral content
BMD: Bone mineral density
GFAP: Glial fibrillary acidic protein
NCAM: Neural cell adhesion molecule
SD: Standard deviation.
